# Supplementary material for: A High-Throughput Method for Quantifying Drosophila Fecundity
Source: Toxics. 2024 Sep 9;12(9):658. doi: 10.3390/toxics12090658 (PMC11436201; doi:10.3390/toxics12090658)
Supplement: Supplementary file 1 [file toxics-12-00658-s001.zip › toxics-3169400-supplementary.pdf]

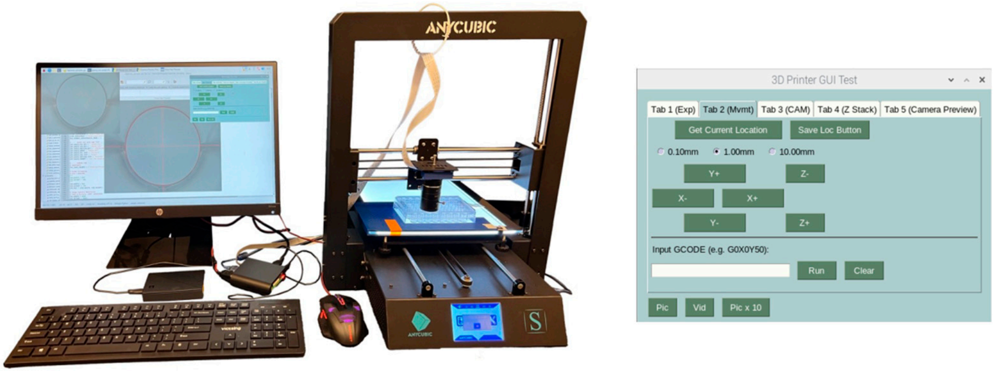

Figure S1. Robocam and graphical user interface.

Photograph of the Robocam setup next to a Raspberry Pi, external hard drive, monitor, mouse, and keyboard. Panel on the right shows the graphical user interface of the software that operates the Robocam.

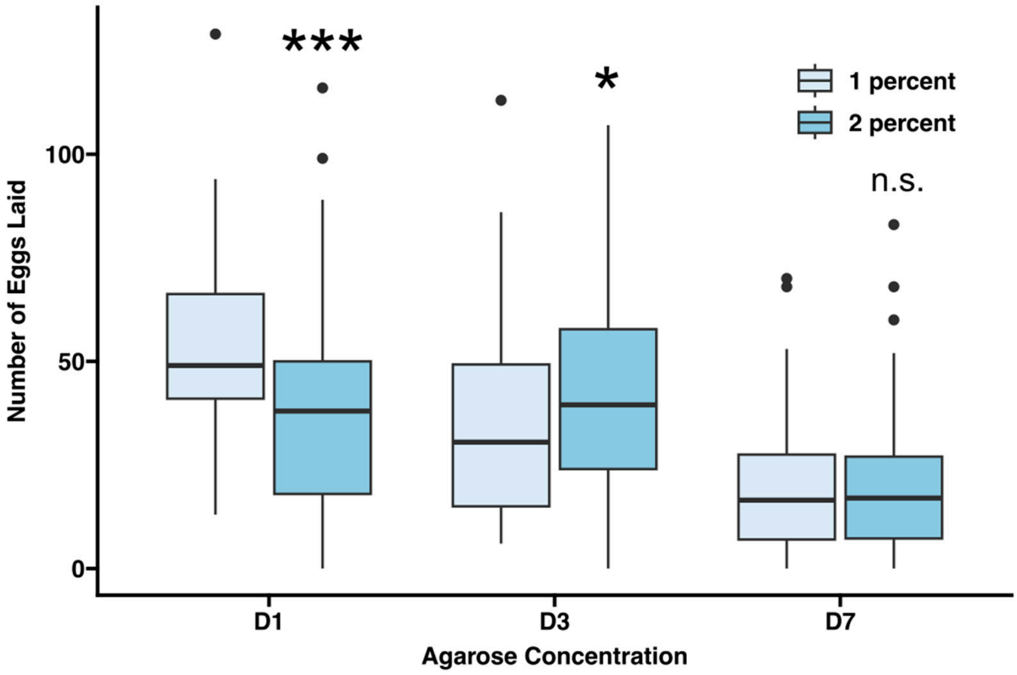

Figure S2. Egg laying rates on media with different concentrations of agarose.

Graph showing the rates of egg laying by *w1118* flies maintained on Grape Juice media with 1% or 2% agarose. Eggs were quantified manually. The data for the 2% agarose condition are taken from Figure 2E. Sample sizes per condition range from 78-133 wells and the data were collected from 3-7 independent replicates. Asterisks indicate statistical significance using Bonferroni-corrected pairwise t-tests between 1 percent and 2 percent for each time point. n.s.: not significant, * $p < 0.05$, *** $p < 0.001$.

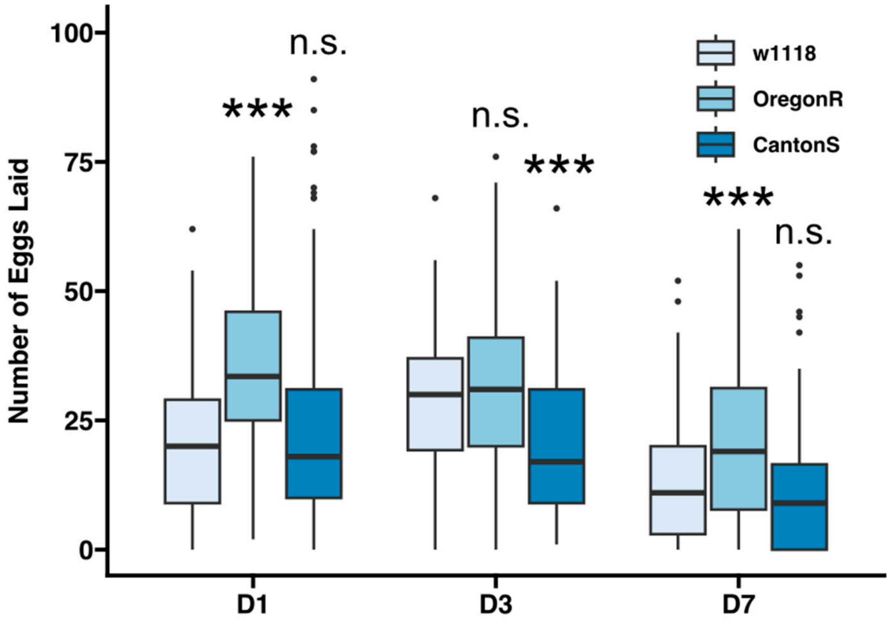

Figure S3. Egg laying rates of three common control strains.

Graph showing the rates of egg laying by *w1118*, *OregonR*, and *CantonS* flies maintained on Grape Juice media with 2% agarose. Sample sizes per condition range from 95-142 wells and the data were collected from 2-3 independent replicates. Asterisks indicate statistical significance using Bonferroni-corrected pairwise t-tests between *w1118* and *OregonR* or *CantonS* for each time point. n.s.: not significant, *** $p < 0.001$.
